# Prevention of oral and maxillofacial trauma secondary to orofacial dyskinesias associated with anti-*N*-methyl-d-aspartate receptor encephalitis: a case series

**DOI:** 10.1186/s12903-021-01783-x

**Published:** 2021-10-10

**Authors:** Jeffrey W. Chadwick, Patricia J. Brooks, Jeffrey M. Singh, David K. Lam

**Affiliations:** 1 Department of Dental Oncology and Maxillofacial Prosthetics, Princess Margaret Cancer Centre, University Health Network, 610 University Avenue, Room 2-933, Toronto, ON M5G 2M9 Canada; 2 Division of Oral and Maxillofacial Surgery, Faculty of Dentistry, University of Toronto, 124 Edward Street, Toronto, ON M5G 1G6 Canada; 3 Medical-Surgical and Neuro-Intensive Care Unit, Toronto Western Hospital, University Health Network, 399 Bathurst St, Toronto, ON M5T 2S8 Canada; 4 Department of Oral and Maxillofacial Surgery, University of the Pacific, Arthur A. Dugoni School of Dentistry, 155 Fifth Street, San Francisco, CA 94103 USA

**Keywords:** Nervous system disease, Central nervous system, Paraneoplastic syndromes, Lingual-facial-buccal dyskinesia, Jaw fixation, Occlusal splint, Oral and maxillofacial surgery, Psychosis, Teratoma

## Abstract

**Background:**

Anti-*N*-methyl-d-aspartate receptor encephalitis (anti-NMDARE) is a multi-stage autoimmune-mediated disease associated with a multitude of neuropsychiatric and dysautonomic features. Orofacial dyskinesias are frequently associated with this condition and manifest as abnormal movements of the orofacial musculature. These involuntary movements may result in significant trauma to the oral and maxillofacial complex including the avulsion of the dentition and orofacial lacerations.

**Case presentation:**

We describe the course of two female patients with anti-NMDARE in whom significant involuntary self-inflicted maxillofacial trauma was suffered despite the use of complex parenteral sedation regimens. The application of traditional maxillomandibular wiring techniques and pharmacologic strategies, including botulinum toxin, to immobilize the mandible were initially unsuccessful. These difficulties led to the fabrication and wire-based fixation of a patient-specific acrylic oral appliance that maintained the mandible in a depressed position and mitigated all lateral and protrusive movements.

**Discussion and conclusions:**

These cases illustrate the first known successful use of an appliance-based therapy for managing orofacial dyskinesias in the anti-NMDARE patient population through an adaptation of traditional maxillomandibular fixation techniques. This approach eliminated further orofacial trauma and afforded physicians with safer means to manage and assess patients afflicted with this condition during their protracted intensive care unit admissions.

## Background

Initially described as the presence of teratomas in a clinical setting of psychiatric syndromes in 2005, anti-*N*-methyl-d-aspartate receptor encephalitis (anti-NMDARE) is a multistage autoimmune disorder primarily affecting adolescents and young adults [1]. The prevalence of this condition is unknown, with an approximate female to male ratio in the order of 4:1 [2]. Anti-NMDARE is associated with significant psychiatric, cognitive, dysautonomic and dyskinetic features which may or may not occur in the presence of neoplastic entities including ovarian and testicular teratomas, seminomas and small cell lung cancer [3]. The *N*-methyl-d-aspartate (NMDA) receptor, composed of NR1 and NR2 subunits that bind glycine and glutamate, respectively, is critical in normal neural network formation and synaptic plasticity [4]. The mechanism by which anti-NMDARE manifests is through immunoglobulin G_1_ (IgG_1_) antibody crosslinking of the NR1 subunit which leads to the internalization of the receptor and its depletion from the synapse [5]. In the early stages of this disease, patients may present with non-specific prodromal symptoms including psychosis, anxiety and delusions [6]. In progressive cases, development of catatonic behavior, orofacial and lingual dyskinesias and changes in level of consciousness may ensue and ultimately lead to erroneous psychiatric diagnoses. These symptoms can be so dramatic that anti-NMDARE is suspected of being an underlying cause for historical accounts of demonic possession [7]. In later stages, anti-NMDARE may manifest by pronounced autonomic instability requiring mechanical ventilation and prolonged intensive care unit (ICU) admissions [8]. T2-weighted and fluid-attenuated inversion recovery (FLAIR) magnetic resonance imaging (MRI) of the brain demonstrate non-specific hyperintense lesions involving multiple cortical and subcortical regions or leptomeningeal enhancement in less than half of all patients diagnosed with anti-NMDARE [9, 10]. Electroencephalogram (EEG) abnormalities are generally characterized by non-specific findings for encephalopathy in 90% of those afflicted with this condition [2, 11]. In some cases, an extreme delta brush pattern may be noted on EEG examination which has been touted as a pathognomonic finding that heralds a poor long-term prognosis [12]. Ultimately, however, the definitive diagnosis of anti-NMDARE is confirmed by the detection of IgG_1_ antibodies in cerebrospinal fluid (CSF) or serum [13, 14]. Unfortunately, diagnosis through antibody detection can be delayed due to extended laboratory turnaround times which may result in deleterious clinical outcomes and poor neurologic recovery secondary to delayed therapy.

During protracted hospital admissions in the setting of anti-NMDARE, significant oral and maxillofacial trauma may occur as a result of orofacial dyskinesias. These injuries include complex intraoral and facial lacerations and tooth avulsion which may result in significant hemorrhage and wound infection. To date, there is a scarcity of literature describing an effective and reliable management strategy for orofacial dyskinesias in this patient population. While other dyskinetic manifestations of this process are potentially mitigated with a variety of pharmacologic agents, there is only one case report describing the successful management of orofacial dyskinesias in a single patient with anti-NMDARE using botulinum neurotoxin serotype A (BoNT-A) [15]. Unfortunately, the potential for the failure of BoNT-A due to insufficient dosing, inadequate target selection amongst the major and accessory muscles of mastication, sensitization due to prior BoNT-A treatment and decreased potency due to issues related to BoNT-A storage or handling can be particularly detrimental in this patient population.

This paper describes the use of a novel oral appliance designed to manage and prevent recurrent oral and maxillofacial trauma resulting from uncontrolled orofacial dyskinesias in patients diagnosed with anti-NMDARE. This appliance is rapidly fabricated and can be effectively utilized for extended periods of time during prolonged ICU admissions.

### Oral appliance design

**Fig. 1:**
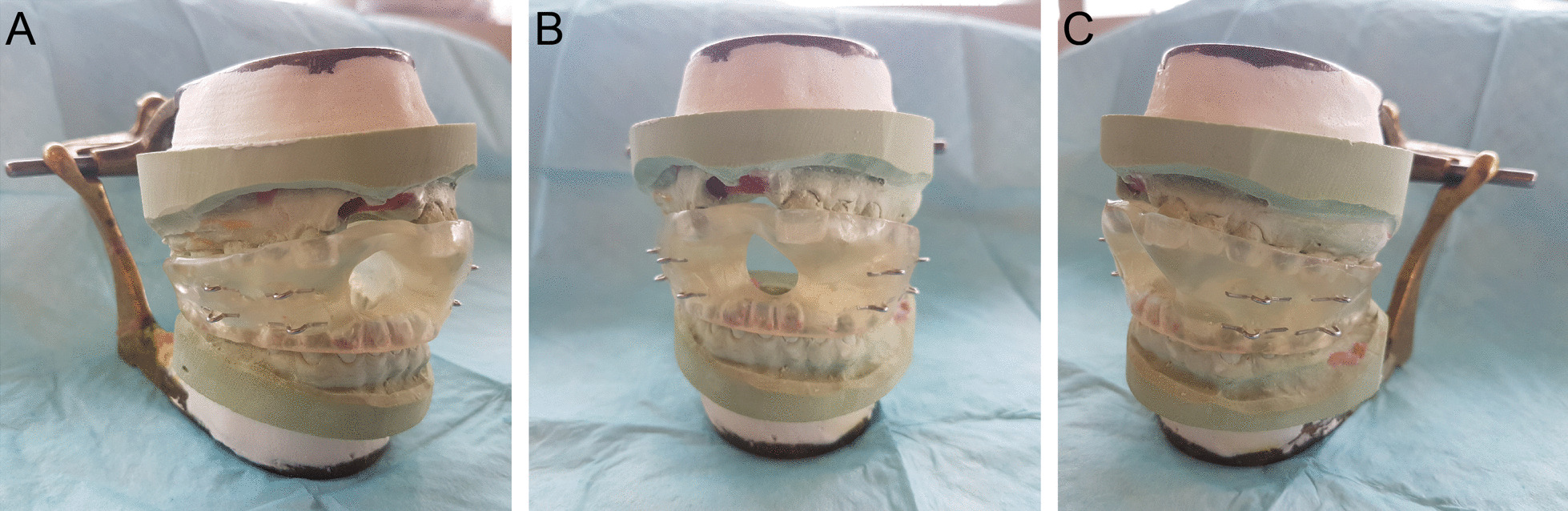
Right lateral oblique (**a**), frontal (**b**) and left lateral oblique (**c**) images of the intraoral appliance on mounted study models

To mitigate the need for increasingly complex anesthetic regimens and to prevent further oral and maxillofacial trauma, an oral appliance with several mechanical and functional design features was constructed to manage orofacial dyskinesias secondary to anti-NMDARE (Fig. 1a–c). The appliance was designed to hold the mandible in a depressed position with a distance between the maxillary and mandibular dentition of greater than approximately 20  millimeters (mm) anteriorly or 11 mm posteriorly. These values were selected based on the position in which the mandible would be placed at the largest mechanical disadvantage as demonstrated in previous masticatory muscle electromyographic and bite force studies [16, 17]. This feature was crucial in reducing the force transmitted to the dentition caused by the dyskinetic gnathic movements. This design also allowed the inclusion of an anterior aperture large enough to facilitate the inspection and cleansing of the oral cavity as well as suctioning of the oropharynx when required. The appliance was fixed to the patient through its ligation between metal loops embedded within the acrylic to maxillary and mandibular Erich arch bars. The arch bars were secured to the existing dentition utilizing interdental wires, thereby distributing all applied gnathic forces to the entirety of both dental arches. The metal loops of the appliance were mechanically engaged into the acrylic in a manner which would not permit their avulsion with the application of continuous isometric forces. The appliance was constructed from an inert acrylic material which intimately engaged approximately 75% of the length of the clinical crowns of the available dentition, limiting the opportunity for the patient to dislodge the appliance with lateral and protrusive mandibular movements. This feature also provided sufficient space for the installation of Erich arch bars and access to perform oral hygiene practices. In order to facilitate the acquisition of maxillary and mandibular alginate impressions for appliance construction as well as its installation in a safe and efficient manner for both the oral and maxillofacial surgeons and patients, general anesthesia with paralysis was induced and monitored by the attending ICU physicians. At the time of appliance installation, patients were transitioned from oral endotracheal tubes to tracheostomies due to the anticipated need for long-term mechanical ventilation.

## Case presentations

We have effectively used this device in five female anti-NMDARE patients ranging from 23 to 34 years of age for between three and  eight months duration who were at risk for significant oral and maxillofacial trauma secondary to orofacial dyskinesias. The described appliance was simple to manufacture and rapidly installed utilizing methods germane to any oral and maxillofacial surgery (OMFS) service. The application of this device affords safer patient management and assessment in conjunction with sedative medications by critical care physicians. Furthermore, its installation mitigates further orofacial trauma thereby decreasing the potential for oral and maxillofacial wound infection and bleeding episodes while eliminating the need for increasingly complex prosthetic rehabilitation following recovery. Below, we discuss two representative examples which chronicle the clinical course of the initial patients affected by anti-NMDARE who were referred to our OMFS service.

### Case 1

A 23-year-old nulliparous woman with a past medical history significant for mild asthma initially presented to a peripheral community hospital in an agitated state with manic symptoms. She was treated for a psychiatric disorder with haloperidol, olanzapine and electroconvulsive therapy (ECT) without any improvement. The patient developed fever, rigidity and tongue-biting movements and was intubated and sedated shortly after admission. A lumbar puncture (LP) was performed revealing the presence of anti-NMDA receptor antibodies confirming the diagnosis of anti-NMDARE. MRI as well as computed tomography (CT) and positron emission tomography (PET) of the head demonstrated no discernable abnormalities. Multiple EEG examinations revealed an extreme delta brush pattern. Screening ultrasound (US) in addition to CT and MRI studies of the thorax, abdomen and pelvis failed to reveal the presence of a teratoma. A trial of intravenous immunoglobulin (IVIG) and methylprednisolone did not result in improvement and the patient was transferred to the ICU at the Toronto Western Hospital (TWH) approximately one month following her initial hospital presentation. Seven cycles of plasma exchange (PLEX) and three cycles of rituximab were administered without any change in patient symptomatology or EEG findings. A tracheostomy and percutaneous endoscopic gastrostomy (PEG) were performed in anticipation for prolonged ventilatory support and ICU admission. Due to refractory symptoms, the patient underwent a bilateral laparoscopic salpingo-oophorectomy, revealing the presence of a mature cystic teratoma associated with the right ovary. Several pharmacologic regimens were unsuccessful in suppressing the patient’s severe dyskinetic movements involving the orofacial region and extremities including tetrabenazine, quetiapine, haloperidol, olanzapine, botulinum toxin deposited within the masseter muscles, and deep sedation with propofol and hydromorphone. Multiple complications were encountered during the patient’s admission including a coagulase-negative staphylococcal bacteremia and ventilator-associated pneumonia.

**Fig. 2:**
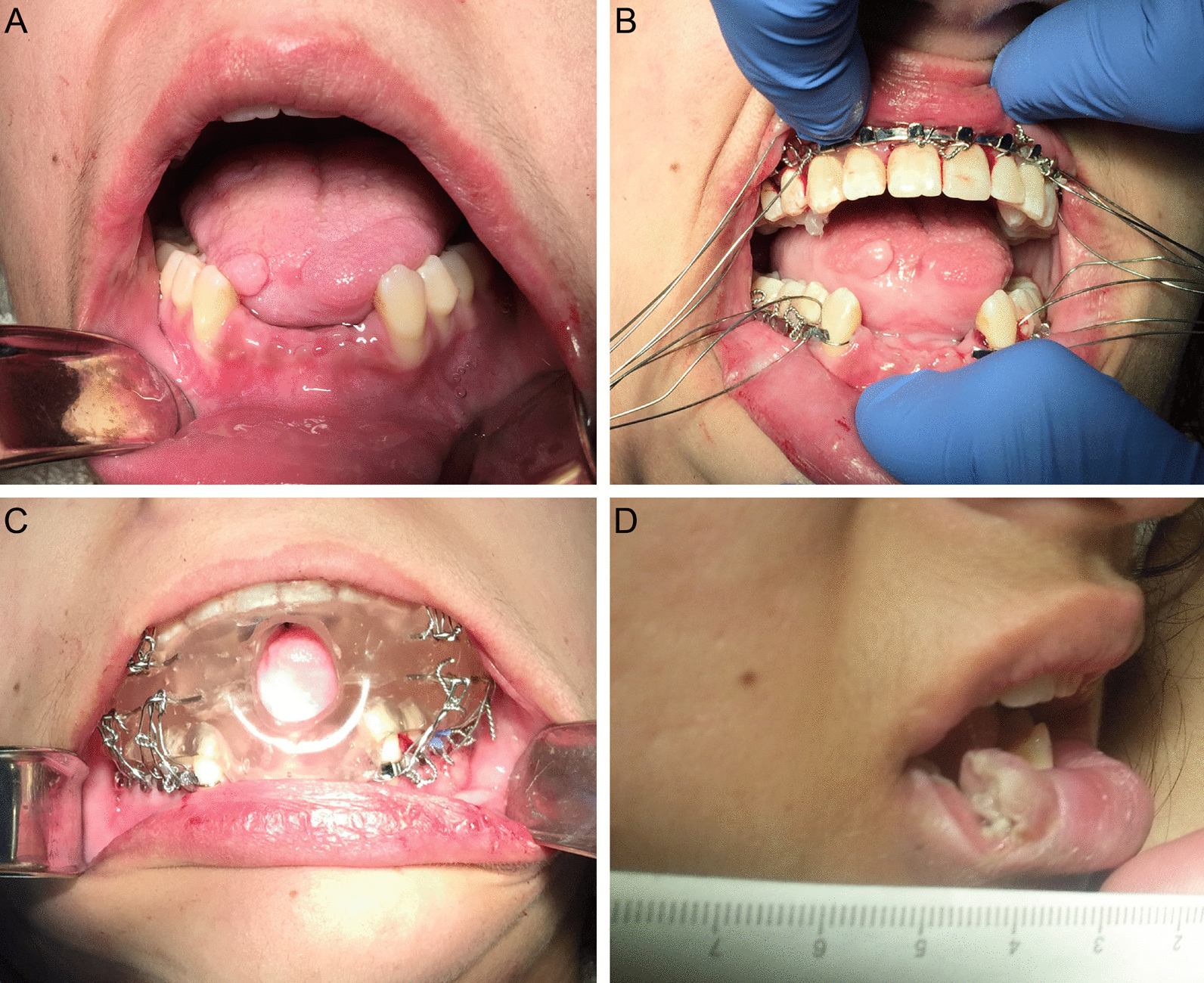
Clinical photograph (**a**) demonstrating the loss of mandibular central and lateral incisors and right anterior tongue trauma two weeks following the initial orofacial dyskinesia-induced trauma. Clinical photograph (**b**) demonstrating the installation of continuous maxillary and interrupted mandibular Erich arch bars with wires prepared for the ligation of the intraoral appliance. Clinical photograph (**c**) of the installed intraoral appliance. Clinical photograph (*d*) of an orofacial dyskinesia-induced full-thickness laceration of the right lower lip following the removal of the appliance

One major complication during this admission was an episode of significant oropharyngeal bleeding. This event occurred secondary to uncontrolled orofacial dyskinesia-induced trauma involving the avulsion of the mandibular central and lateral incisors and a significant laceration of the right anterior tongue which required the transfusion of packed red blood cells (Fig. 2a). To prevent further trauma, maxillomandibular fixation (MMF) was attempted utilizing several techniques including the application of Ivy loops, Ernst ligatures and Erich arch bars. These approaches all failed due to her continued and worsening orofacial dyskinesias, despite the later administration of BoNT-A to the masseter muscles. Our oral appliance was subsequently fabricated which placed the mandible in a depressed position and was secured via 24-gauge steel ligatures to previously installed maxillary and mandibular Erich arch bars that were placed during the initial attempts at MMF. Following the application of this appliance, the patient suffered no further orofacial trauma (Fig. 2b, c). After eight months, the oral appliance was removed due to improvement in bodily dyskinetic movements. Unfortunately, the patient still possessed significant orofacial dyskinesias and, now able to express a full range of mandibular movement, lacerated her right lower lip in a full-thickness fashion (Fig. 2d). Two months later, the patient had improved significantly, demonstrating an increased level of alertness and responsiveness without the presence of dystonic movements. She was subsequently transferred to the neurology ward in stable condition for continued convalescence where no further intervention or follow-up was required by the OMFS service.

### Case 2

**Fig. 3:**
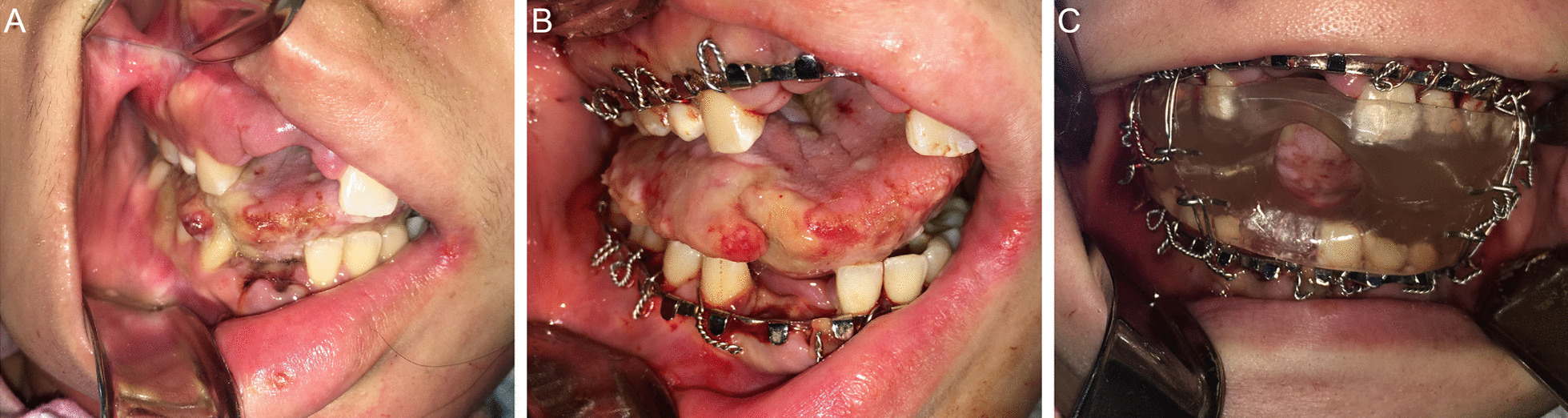
Clinical photograph (**a**) demonstrating the loss of the right maxillary and mandibular central and lateral incisors and right anterior tongue laceration following orofacial dyskinesia-induced trauma. Clinical photograph (**b**) demonstrating the installation of continuous maxillary and mandibular Erich arch bars. Clinical photograph (**c**) of the installed intraoral appliance

**Fig. 4:**
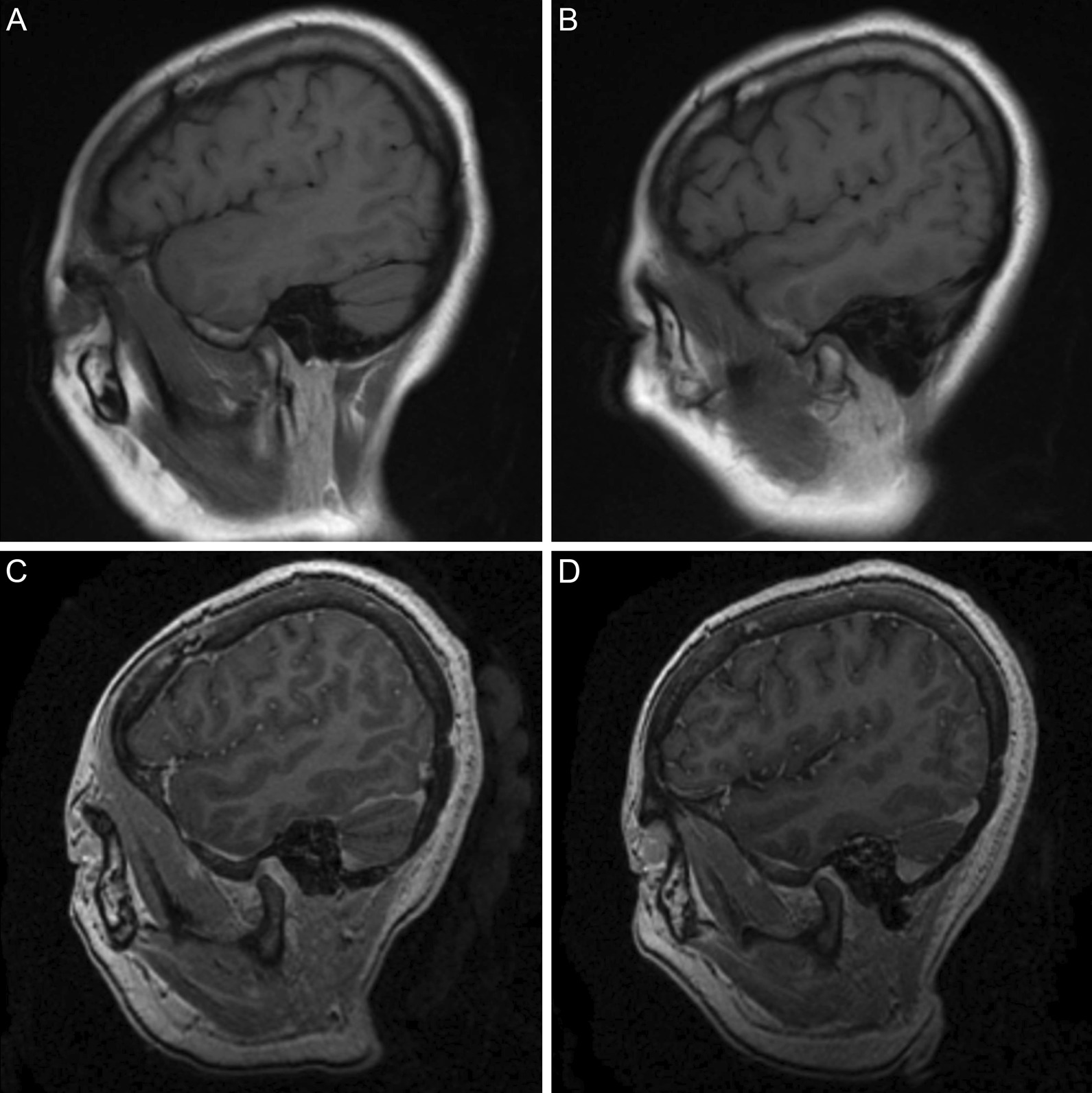
Selected T1-weighted (**a**, **b**) and contrast-enhanced three-dimensional fast spoiled gradient-echo (**c**, **d**) magnetic resonance imaging of the left (**a**, **c**) and right (**b**, **d**) temporomandibular joints demonstrating normal osseous anatomy and articular disc position

A previously healthy nulliparous 25-year-old woman presented to a peripheral community hospital with new-onset right-sided twitching, generalized tonic–clonic seizures, confusion and hallucinations. CT imaging of the head was negative for structural abnormalities, while US imaging of the pelvis revealed a mass associated with the left ovary. Initial treatment with phenytoin, propofol, valproic acid and clobazam was ineffective and necessitated patient transfer to TWH. Upon arrival, the patient exhibited  irregular movement of all limbs and non-purposeful eye movements as well as lip smacking and chewing motions which were initially managed with clonazepam and clonidine. CT and MRI studies of the brain did not reveal structural lesions nor parenchymal, leptomeningeal or dural enhancement. The patient was intubated and, shortly thereafter, luxated the right maxillary and mandibular central and lateral incisors resulting in marked hemorrhage requiring consultation with the OMFS service (Fig. 3a). After the extraction of the extremely mobile dentition, a tracheostomy was performed with installation of our oral appliance to mitigate further oral and maxillofacial trauma (Fig. 3b, c). Repeat US demonstrated a 1.8 centimeter (cm) × 1.8 cm × 1.8 cm hypoechoic lesion associated with the left ovary suspicious for an ovarian teratoma. Anti-NMDA receptor antibodies were demonstrated within the CSF confirming the diagnosis of anti-NMDARE. The patient underwent a left laparoscopic salpingo-oophorectomy with histopathology revealing a mature teratoma associated with the left ovary. Unfortunately, the patient continued to deteriorate despite medical and surgical management including several courses of IVIG, PLEX, intravenous steroids, rituximab and a laparoscopic right salpingo-oophorectomy. Histopathology noted the presence of several cystic follicles and a benign simple cyst with no identifiable neural tissue or teratoma associated with the right ovary. Three months following her original hospital admission, dyskinetic movements and seizures terminated and the patient began to breathe spontaneously. The oral appliance was subsequently removed and the patient was eventually transferred to the neurology ward in stable condition where no further intervention or follow-up by the OMFS service was required. A review of the discharge MRI which captured the temporomandibular joint (TMJ) regions revealed an absence of osseous degeneration of the articulating components of the joints and no discernible disc abnormalities secondary to prolonged oral appliance use (Fig. 4a–d).

## Discussion

Patients diagnosed with anti-NMDARE often suffer from significant involuntary self-inflicted maxillofacial trauma secondary to severe orofacial dyskinesias. We describe the application of a novel oral appliance to manage orofacial dyskinesias in anti-NMDARE patients with several custom mechanical and functional design features. While we demonstrate the efficacy of this appliance in reducing the incidence of oral and maxillofacial trauma, it is not a replacement, but rather, an adjunct for the judicious use of sedative medications to mitigate ataxic movement in this patient population during their hospitalization.

Orofacial dyskinesias are regarded as a characteristic clinical presentation of anti-NMDARE that can result in significant morbidity if not adequately controlled [13, 18]. Despite aggressive supportive care, immunosuppressive agents and tumor resection, many patients with severe disease have prolonged ICU admissions and may require complex sedation regimens to suppress dangerous or injurious movements [1]. In such cases, prolonged use of high-dose sedatives such as propofol, benzodiazepines and ketamine have adverse effects on hemodynamics and respiratory function and may place patients at increased risk of infection and critical illness myopathy. In the setting of anti-NMDARE, the NMDA receptor is internalized in response to the binding of anti-NMDA receptor antibodies. Propofol functions through activation of the gamma-aminobutyric acid (GABA) receptor as well as inhibition of the NR1 subunit of the NMDA receptor and its action can be unpredictable with large and continuous doses in patients afflicted with anti-NMDARE [19]. Ketamine is a non-competitive antagonist of the NMDA receptor and may have unpredictable physiological effects on respiration and hemodynamics in this already fragile patient cohort [20]. Finally, there is the potential to induce or worsen orofacial dyskinesias in the anti-NMDARE patient population when treatment with anticonvulsant medications is required [21, 22].

Despite the prolonged installation of an oral appliance which drastically altered the vertical dimension of occlusion (VDO), clinical assessment of patients treated in this fashion during their consultation for oral rehabilitation after discharge revealed no evidence of TMJ internal derangement. Currently, there is a dearth of well-designed studies which investigate the effect of large changes in VDO and the development of symptoms suggestive of temporomandibular dysfunction (TMD). More subtle changes in VDO have, however, resulted in only transient and mild discomfort in the TMJ region which was unlikely in these cases as our oral appliance was designed in a manner which promoted maximum occlusal stability through significant coronal contact [23].

Classic wire-based MMF techniques were initially ineffective in establishing occlusal stabilization as they could not completely terminate lateral and protrusive movements in this patient population despite the presence of the majority of their dentition and a stable occlusion. The authors recognize that other methods for occlusal stabilization are available and may have included the use of intermaxillary fixation (IMF) screws or cementation of vacuum-formed splints. Unfortunately, in the setting of maxillofacial trauma, IMF screw failure is generally noted after approximately one month posing a considerable aspiration risk and cement-retained vacuum-formed splints are unable to adequately immobilize unstable mandibular fractures [24, 25]. As such, it was anticipated that these MMF treatment options would have quickly failed in this patient population due to the severity of the dyskinetic jaw movements, potentially resulting in further oral and maxillofacial trauma. While these techniques are both technically simple and allow for rapid means of establishing MMF, their touted advantages over wire-based techniques related to oral hygiene maintenance and wire-related complications are contentious in these cases [26, 27]. First, oral hygiene was maintained via chlorhexidine swabbing as well as the avoidance of an oral diet due to the necessity for PEG feeding [28]. Second, injuries to the periodontium caused by interdental wires have been regarded as both temporary and reversible [29]. Third, as in our cases, injury to the clinicians applying interdental wires was mitigated through the practices of double gloving and the use of local anesthetics, deep sedation and, when required, short-acting paralytics [26]. Furthermore, with the use of our MMF technique, ICU staff were comfortable in being tasked to release the intermaxillary fixation wires with a set of wire cutters which were affixed to the head of the bed in the event that prompt removal of the appliance was required. In emergency situations, the removal of a cement-retained appliance may have proved both cumbersome and difficult when attempted by non-dental personnel. Finally, the authors caution against the use of common dental bite blocks as well as other devices fabricated from tongue depressors. These devices cannot be rigidly affixed to the dentition which may result in their full or partial expulsion from the oral cavity and cause damage to oral soft tissues or dentition, be ingested or act as a choking hazard.

While the advent of this appliance in relation to managing orofacial dyskinesias in anti-NMDARE patients may appear extraneous given the myriad of critical medical complications associated with this disease process, its importance cannot be overstated. The placement of our oral appliance eliminated the need for management of ingested or aspirated teeth, infection or sepsis, or bleeding episodes requiring transfusion secondary to involuntary self-inflicted oral and maxillofacial trauma. The use of this appliance also afforded a copacetic clinical appearance, easing the anxiety of visiting family members caused by the otherwise alarming degree of dyskinetic jaw activity, especially in those cases where orofacial trauma had already manifested. Overall, the clinical benefit of the appliance allowed for transient weaning of sedative medications to facilitate patient assessment following both medical and surgical therapy without risk of further oral and maxillofacial trauma during lengthy hospitalizations. Our limited experience with this patient population cannot be directly extrapolated to all patients with oral dyskinesias. However, in conjunction with the support of an ICU and immunotherapy with or without tumor ablation, oral appliance therapy was critical for a positive outcome in these patients with significant orofacial dyskinesias secondary to anti-NMDARE.

## Data Availability

Not applicable.

## Abbreviations

anti-NMDARE: Anti-*N*-methyl-d-aspartate receptor encephalitis
BoNT-A: Botulinum neurotoxin serotype A
cm: Centimeters
CSF: Cerebrospinal fluid
CT: Computed tomography
ECT: Electroconvulsive therapy
EEG: Electroencephalogram
FLAIR: Fluid-attenuated inversion recovery
GABA: Gamma-aminobutyric acid
IgG_1_: Immunoglobulin G_1_
ICU: Intensive care unit
IMF: Intermaxillary fixation
IVIG: Intravenous immunoglobulin
LP: Lumbar puncture
mm: Millimeters
MMF: Maxillomandibular fixation
MRI: Magnetic resonance imaging
NMDA: *N*-Methyl-d-aspartate
OMFS: Oral and maxillofacial surgery
PEG: Percutaneous endoscopic gastrostomy
PET: Positron emission tomography
PLEX: Plasma exchange
TMD: Temporomandibular dysfunction
TMJ: Temporomandibular joint
TWH: Toronto Western Hospital
US: Ultrasound
VDO: Vertical dimension of occlusion
